# Dissemination and Genetic Relatedness of Multidrug-Resistant and Extensively Drug-Resistant *Acinetobacter baumannii* Isolates from a Burn Hospital in Iraq

**DOI:** 10.1155/2022/8243192

**Published:** 2022-05-27

**Authors:** Aras A. K Shali, Paywast J Jalal, Sehand K Arif

**Affiliations:** Dept of Biology, College of Science, University of Sulaimani, Al Sulaymaniyah, Iraq

## Abstract

*Acinetobacter baumannii* is an aggressive opportunistic bacterial pathogen that causes severe nosocomial infections, especially among burn patients. An increasing number of hospitals-acquired infections have been reported all over the world. However, little attention has been paid to the relatedness between *A. baumannii* isolates from different hospital environments and patients. In this study, 27 isolates were collected from the Burn and Plastic Surgery Hospital of Al Sulaymaniyah City, Iraq, from January through December 2019 (11 from patients and 16 from the wards environment), identified to species level as *A. baumannii* using Vitek 2 system and molecular detection of *16S rRNA* gene, and then confirmed by targeting the *bla*_OXA-51_ gene. Moreover, the isolates were characterized by means of automated antimicrobial susceptibility assay, antimicrobial-resistant patterns, a phenotypic method using a combined disk test, and molecular methods for the detection of class A and C *β*-lactamase genes, and finally, the genetic relatedness was classified. Antimicrobial susceptibility testing showed that 63% (17/27) of the retrieved *A. baumannii* isolates were extensively drug-resistant to 8/9 antimicrobial classes. Furthermore, 37% (10/27) of the isolates were classified as multidrug-resistant; 8 isolates exhibited similar resistant patterns and the other two isolates showed 2 different patterns, while resistance was greater in isolates from patients than from the ward environment. Combined disk test showed that two isolates contained extended-spectrum *β*-lactamase. All isolates carried *bla*_TEM-1_, and two copies of the *bla*_CTX-1_ gene were indicated in one isolate, while *bla*_SHV_ was absent in all isolates. Twenty-four isolates carried the *bla*_AmpC_ gene; among them, 3 isolates harbored the insertion sequence IS*Aba-1* upstream to the gene. Using Enterobacterial Repetitive Intergenic Consensus PCR, the isolates were clustered into 6 distinct types; among them, two clusters, each of four strains, were classified to contain isolates from both patients and environments. The clusters of similar genotypes were found in inpatients as well as the environments of different wards during time periods, suggesting transmission within the hospital. Identification of possible infection sources and controlling the transmission of these aggressive resistance strains should be strictly conducted.

## 1. Introduction

Multiantibiotic resistance to bacterial pathogens has increased worldwide which is considered a public health hazard. The development of multidrug-resistant MDR and extensively drug-resistant XDR bacteria is reported in several recent studies which require urgencies to upgrade the currently in-use antimicrobial agents or to create new alternatives [1]. Besides, there should be frequent monitoring of the antimicrobial susceptibility testing to indicate the antibiotic of choice and to identify the resistance patterns [2].

*A. baumannii* has over the last several years gained recognition as being an opportunistic pathogen. While usually a benign component for inpatients, it causes about 10% of nosocomial infections in intensive care units (ICUs), leading to a wide range of infections such as bacteremia, secondary meningitis, pneumonia, burns, wounds, and urinary tract infections [3]. Clinically, its significance has been forced by its capacity to acquire resistance determinants which makes it a highly threatening organism within the field of antibiotics. *A. baumannii* has high intrinsic resistance to several antibiotics and a tendency to acquire resistance genes that rise its antimicrobial resistance patterns [4]. Permeability defects, synthesis of numerous *β*-lactamases (extended-spectrum *β*-lactamase (ESBL), oxacillinase (OXA), and metallo-*β*-lactamase (MBL) types), overexpression of intrinsic *β*-lactamases, and efflux pumps are found to be major resistance mechanisms exhibited by *A. baumannii* [5]. Most *A. baumannii* strains have become progressively resistant to nearly all antibiotics that are currently available due to the existence of mobile genetic elements, such as plasmids and insertion sequences [6, 7]. Furthermore, the transmission of these determinants between chromosomes and plasmids may participate in the dissemination of resistance among different bacterial strains. Increased rates of antibiotic resistance in *A. baumannii* strains are of significant importance in health settings all over the world. Resistance to *β*-lactam drugs is mainly attributed to ESBLs, oxacillinases, and MBLs production [8]. ESBL producers are mutants, acquired plasmids that mediate *β*-lactamases, which exhibit unique hydrolytic properties (with unique properties of hydrolysis). These enzymes have the capacity to hydrolyze a wide range of *β*-lactam drugs such as third-generation cephalosporins and monobactams [3]. Class C *β*-lactamases are cephalosporinases (AmpC) that are chromosomally encoded and naturally produced by all *A. baumannii* strains [9]. The most common and frequent types of ESBL genes include *bla*_SHV_, *bla*_TEM_, and *bla*_CTX-M_; they are associated with mobile genetic elements, predominately plasmids [7, 10]. The dissemination of multidrug-resistant *A. baumannii* in both hospital wards and inpatients is of great epidemiologic importance [11]. Genotyping methods are of essential role in outbreak investigations for epidemiological classifications in nosocomial infections; the most simple, convenient, and common method used for genotyping is enterobacterial repetitive intergenic consensus PCR (ERIC-PCR) [12-14].

This study aimed to identify and characterize *A. baumannii* isolates from inpatients and environments in Burn and Plastic Surgery Hospital, by means of their antimicrobial resistance pattern and phenotypic and genotypic detection of ESBL-producing isolates, as well as identifying multidrug, extensively drug, and pan-drug resistant strains, and to find out genetic relatedness between strains that were isolated from the ward environment and inpatients in a burn hospital using ERIC-PCR.

## 2. Materials and Methods

### 2.1. Bacterial Isolation and Identification

Fourteen bacterial isolates from burn inpatients in different wards (coded P) previously identified as *A. baumannii* were collected from the Burn and Plastic Surgery Hospital during the period from January to December 2019.

Environmental samples (coded E) were taken in different sites at the critical burn unit, adult and pediatric wards, operation halls, toilets and bathrooms, and outpatient department in the same hospital and time frame mentioned above and targeted to the most critical and representative locations in each department. One hundred random, undirected swabs were taken from inanimate surfaces and equipment, including beds, walls, monitors, ventilators, bedside tables, operation tables, anesthesia equipment, trolleys, benches, stainless steel IV stands, medicine cabinets, critical burn unit office tables, chairs, doors knobs, bins, and surgical blades. All samples were then cultured on suitable bacteriological media for the search for *A. baumannii* [15]. The swabs were cultured in Tryptone Soy Broth (Neogen, UK), incubated at 37^o^C overnight, then streaked on MacConkey agar plates (Neogen, UK), and incubated at 37 °C for 18–24 hours for the selection of Gram-negative bacteria [15]. Suspected colonies were selected for presumptive tests by growing on MacConkey agar, colony morphology, oxidase reaction (Hach, USA), catalase test, and Gram stain [1].

Bacterial identities were identified to species level using Vitek 2 GN ID Card system according to the manufacturer's recommendations (BioMérieux, France), the 16S rRNA gene [16] and confirmed by targeting *bla*_OXA-51_ gene [17]. *A. baumannii* ATCC 19606 and *Escherichia coli* ATCC 25922 were used as the reference strains.

### 2.2. Antimicrobial Susceptibility Testing

Vitek 2 system was used to test antimicrobial susceptibility according to the manufacturer's guidelines (BioMérieux, France). The following antimicrobial classes were tested: penicillins (penicillin and piperacillin), *β*-lactam-*β*-lactamase inhibitor (ampicillin-sulbactam), carbapenem (meropenem, cephalosporins; cefotaxime, ceftazidime, cefazolin, cefoxitin, and cefepime), sulfonamides (trimethoprim/sulfamethoxazole), aminoglycosides (gentamicin and tobramycin), fluoroquinolones (levofloxacin and ciprofloxacin), nitrofurans (nitrofurantoin and tetracyclines), and minocycline. The phenotypic resistance patterns of the isolates were classified into multidrug resistance (MDR), extensively drug resistance (XDR), and pan-drug resistance (PDR) according to CLSI [18].

### 2.3. Phenotypic Detection of ESBL-Producing Isolates

A combined disk test (CDT) screening method was used for the phenotypic identiﬁcation of ESBL-producing isolates. Antibiogram disks (Bio-Rad, USA) containing cefotaxime CTX (30 *μ*g), ceftazidime CAZ (30 *μ*g), and cefepime FEP (30 *μ*g) alone and in combination with clavulanic acid (10 *μ*g) were used. According to the CLSI standards, an increase in resistant zone diameter of ≥5 mm in the presence of clavulanic acid indicates the existence of ESBL in the test organism [18].

### 2.4. Molecular Detection of ESBL Genes, Class A and Class C

Four ESBL genes were selected in this study to detect *bla*_TEM-1_, *bla*_SHV_, *bla*_CTX-M-1_ [19], and *bla*_AmpC_ [20] genes by PCR using specific sets of primers (Table 1). Molecular detection of the target genes was carried out on chromosomal and plasmid DNA extracts using Presto Mini gDNA Bacteria Kit (Geneaid Biotech Ltd., Taiwan) and a PureYield™ Plasmid Miniprep System (Promega, USA), respectively [21, 22], following the manufacturer's recommendations, except for *bla*_AmpC_ gene, which was carried out only in chromosomal DNA. Isolates carrying *bla*_AmpC_ gene were further screened to search for insertion sequence IS*Aba-1* located upstream of it [23].

Each amplification reaction in this study was performed in a final volume of 20 *μ*L. Each reaction PCR tube contained 5 *μ*L of the reaction mixture (GeneDirex, Taiwan), 2 *μ*L DNA template, 0.5 *μ*L 10x of each primer (Sinaclone, Iran), and 12 *μ*L ddH_2_O. The PCR amplifications were completed in a Veriti® 96-well thermal cycler (Applied Biosystem, USA). The PCR products were separated using 1.5% agarose gel electrophoresis (Cleaver, Scientific, Ltd, UK) in 1x TAE buffer, 80V for 60 min, followed by staining with 0.5 *μ*g/mL ethidium bromide, and then visualized under ultraviolet illumination using Gel Doc XR+ (Bio-Rad, USA).

### 2.5. Enterobacterial Repetitive Intergenic Consensus PCR

The genetic relationships among the 27 clinical *A. baumannii* isolates were determined by ERIC-PCR [24]. Primers' details are listed in Table 1.

Different DNA patterns were gained by gel electrophoresis and analyzed using GelAnalyzer software. The DNA fingerprints for the gel images were considered by conducting the Dice coefficient. The unweighted pair group method using arithmetic averages (UPGMAs) was applied to construct the phylogenetic tree. The clonal relationship was constructed based on the similarity matrix, and ERIC fingerprints generated a dendrogram among the identities. Isolates in the clustered dendrogram with more than 90% similarity were considered clonally related [13].

### 2.6. Statistical Analyses

The obtained data were analyzed using the chi-square test statistic (GraphPad prism version 9, California, USA) (significance level; *P* < 0.05). A heat-map data analysis was used as a data visualization tool to show the height and width of the graph.

## 3. Results

### 3.1. Phenotypic Characterization of the Recovered *A. baumannii* Isolates

The recovered colonies showed a faint pink color, nonlactose fermenter on MacConkey agar, negative oxidase test, catalase-positive, and pleomorphic Gram-negative bacilli; these colonies were presumptively identified as *Acinetobacter* sp. Eleven isolates from patients and sixteen isolates from the hospital environment were identified to species level as *A. baumannii* using Vitek 2 system, 16S rRNA, and *bla*_OXA-51_ gene. The sources of the isolates are listed in Tables 2 and 3.

**Figure 1:**
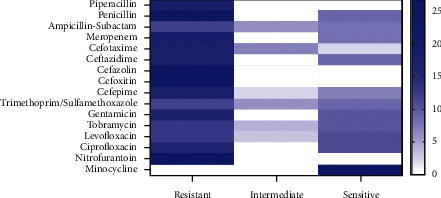
The heat map illustrating the different degrees of susceptibility of the retrieved *A. baumannii* to different antimicrobial agents used in this study.

**Figure 2:**
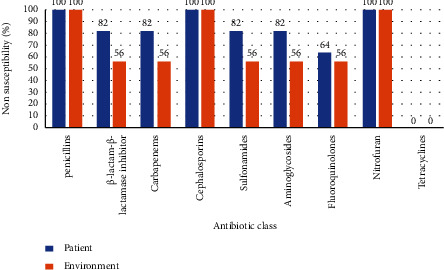
Percentage of nonsusceptibility of *A. baumannii* to different antibiotic classes.

According to the Vitek 2 system, the recovered *A. baumannii* isolates exhibited remarkable resistance patterns to various antimicrobial classes including penicillins: amoxicillin and penicillin (100%), piperacillin (66.6%), *β*-lactam-*β*-lactamase-inhibitor combination: ampicillin-sulbactam (44.4%), carbapenem: meropenem (70.3%), cephalosporins: cefazolin (100%), cefoxitin (100%), cefotaxime (66.6%), ceftazidime (66.6%), and cefepime (66.6%), sulfonamides: trimethoprim-sulfamethoxazole (44.4%), aminoglycosides: gentamicin (62.9%) and tobramycin (48.1%), fluoroquinolones: levofloxacin (48.1%) and ciprofloxacin (59.2%), and nitrofuran: nitrofurantoin (100%). Moreover, the tested isolates displayed intermediate resistance to *β*-lactam-*β*-lactamase-inhibitor combination: ampicillin-sulbactam (22.2%), cephalosporins: cefotaxime (25.9%) and cefepime (7.4%), sulfonamides: trimethoprim-sulfamethoxazole (22.2%), aminoglycosides: tobramycin (14.8%), and fluoroquinolones: levofloxacin (11.1%). Besides, the retrieved isolates were sensitive to penicillins: piperacillin (33.3%), *β*-lactam-*β*-lactamase-inhibitor combination: ampicillin-sulbactam (33.3%), carbapenem: meropenem (29.6%), cephalosporins: cefotaxime (7.4%), ceftazidime (33.3%) and cefepime (25.9%), sulfonamides: trimethoprim-sulfamethoxazole (33.3%), aminoglycosides: gentamicin and tobramycin (37%), fluoroquinolones: levofloxacin and ciprofloxacin (40.7%), and tetracyclines: minocycline (100%) (Table 4, Figure 1). Nonsusceptibility percentages to different antibiotic classes illustrated that resistance was greater in isolates from patients than from the ward environment (Figure 2).

In addition, our findings revealed that 63% (17/27) of the retrieved *A. baumannii* isolates are extensively drug-resistant (XDR: resistant to ≥ one agent in all but ≤ two antimicrobial classes) to 8 antimicrobial classes: penicillins (penicillin and piperacillin), *β*-lactam-*β*-lactamase inhibitor (ampicillin-sulbactam), carbapenem (meropenem), cephalosporins (cefotaxime, ceftazidime, cefazolin, cefoxitin, and cefepime), sulfonamides (trimethoprim/sulfamethoxazole), aminoglycosides (gentamicin and tobramycin), fluoroquinolones (levofloxacin and ciprofloxacin), and nitrofurans (nitrofurantoin), whereas a single isolate showed resistant to 7 antimicrobial classes: penicillins (penicillin and piperacillin), *β*-lactam-*β*-lactamase inhibitor (ampicillin-sulbactam), carbapenem (meropenem), cephalosporins (cefotaxime, ceftazidime, cefazolin, cefoxitin, and cefepime), sulfonamides (trimethoprim/sulfamethoxazole), aminoglycosides (gentamicin and tobramycin), and nitrofurans (nitrofurantoin). Furthermore, the phenotypic resistance patterns of the isolates classified 37% (10/27) of the isolates as multidrug-resistant (MDR: resistant to ≥ one agent in ≥ in 3 antimicrobial classes); 8 isolates exhibited resistance to penicillins (penicillin and piperacillin), cephalosporins (cefotaxime, ceftazidime, cefazolin, cefoxitin, and cefepime), and nitrofurans (nitrofurantoin); the other two isolates showed 2 different resistant patterns: first, penicillins (penicillin and piperacillin), *β*-lactam-*β*-lactamase inhibitor (ampicillin-sulbactam), carbapenem (meropenem and cephalosporins), cefotaxime (ceftazidime, cefazolin, cefoxitin, and cefepime), sulfonamides (trimethoprim/sulfamethoxazole), and ciprofloxacin and nitrofurans (nitrofurantoin) and second penicillins (penicillin and piperacillin), cephalosporins (cefotaxime, ceftazidime, cefazolin, cefoxitin, and cefepime), aminoglycosides (gentamicin and tobramycin), and nitrofurans (nitrofurantoin) (Table 5).

**Figure 3:**
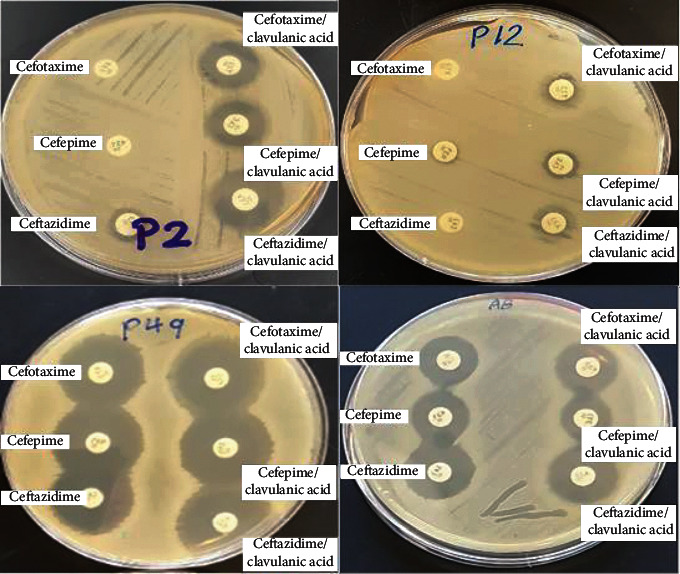
Combined disk test for phenotypic detection of ESBL-producing *A. baumannii.*

Data achieved from the combined disk test (CDT) showed that two isolates, P2 and P12, were ESBL positive among patients' isolates. According to the performed tests for isolate P2, the inhibition zones of ceftazidime, cefotaxime, and cefepime were increased with clavulanic acid combination by 18, 17, and 14 mm, respectively. Isolate P12 shows increased inhibition in zone diameter with clavulanic acid combination by 13, 12, and 5 mm, respectively (Figure 3).

The experiments showed that the *bla*_CTX-M-1_ gene was detected only in isolate P2, in both its genomic and plasmid DNA. Out of the 27 isolates, only one environmental isolate E53 lacked the *bla*_TEM-1_ gene on the chromosomal DNA, while all isolates carried the *bla*_TEM-1_ gene on their plasmids. Isolates carrying the SHV gene were not detected. On chromosomal DNA, the *bla*_AmpC_ gene was detected at a rate of 88.88% (*N* = 24) of the total samples, and 3 patients' isolates lacked the gene. Three isolates were found to harbor insertion sequence IS*Aba-1* upstream of the *bla*_AmpC_ gene from patients' isolates.

In this study, there are direct relations between genotypic and phenotypic resistant patterns in the tested *A. baumannii* isolates; 100% (*N* = 27) of the retrieved strains are resistant to penicillin and cephalosporin classes and carried ESBL genes, *bla*_TEM-1_. Furthermore, twenty-four isolates were found to carry the *bla*_AmpC_ gene, among them, 3 isolates harbored upstream IS*AB-1*, and they showed nonsusceptibility to class cephalosporines (100%) in addition to penicillins.

**Figure 4:**
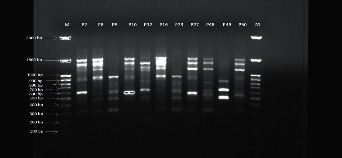
ERIC pattern of *A. baumannii* from patients' isolates.

**Figure 5:**
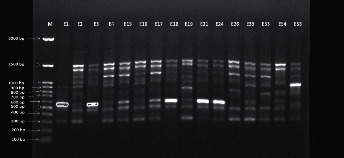
ERIC pattern of *A. baumannii* from environment isolates.

The 27 *A. baumannii* strains were molecularly typed by using the ERIC-PCR technique. Patients' and environment isolates were subjected to electrophoresis. The banding patterns comprised 3 to 12 fragments/strain. The molecular sizes of the bands ranged from 200 to 1500 bp. Patterns with genetic similarity from ≥90% were considered a cluster as shown in Figures 4 and 5.

**Figure 6:**
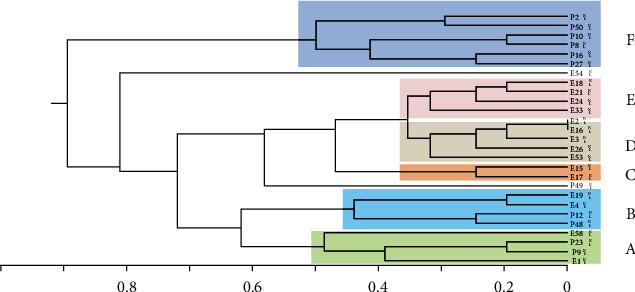
Dendrogram of ERIC-PCR analysis for 27 *A. baumannii*. (a–d) Different clusters.

The ERIC dendrogram analysis by GelAnalyzer software for the 11 patients' and the 16 environmental isolates classified these 27 *A. baumannii* strains into six distinct groups (Table 5). Interestingly, two clusters (A and B), each of four strains, were classified to contain isolates from both patients and environments, as illustrated in Figure 6. Clusters C, *D,* and *E* contained environmental isolates, while cluster *E* contained isolates from patients only (Table 6). Three isolates were assigned to have three different patterns.

## 4. Discussion

*A. baumannii* is a multidrug-resistant bacterium which, due to its survival capacity in hospital milieus and ability to develop nosocomial outbreaks, is a global medical threat [25]. *A. baumannii* uses numerous virulence factors, including porins, enzymes, capsules, cell wall lipopolysaccharide, biofilm production, motility, and iron-acquisition mechanisms. These virulence factors permit the pathogen to resist the strict hospital environment conditions and enable the development of various infections. The increased occurrence of infections caused by *A. baumannii* and the multiplicity of resistance mechanisms adversely affect the majority of antibiotic classes to exhibit minimal effectiveness. A wide range of antibiotic hydrolyzing enzymes such as efflux pump changes, impermeability, and antibiotic target mutations direct *A. baumannii* to maintain as a multidrug-resistant pathogen, with further complicating treatment [26].

The emergence of MDR/XDR pathogens over the last decade is dramatically increasing. Many studies have been conducted to magnify the impact of MDR, XDR, and PDR (pan-drug resistance) on strategies and protocols of treatment, especially nosocomial infections caused by *A. baumannii* [27-29]. Recent developments in the field of antimicrobial drugs are devoted to the use of natural antimicrobial and nanoparticle delivery agents [30].

In this study, molecular detection of *bla*_CTX-M_ and *bla*_TEM-1_ genes may confer resistance to cephalosporins such as ceftazidime, cefazolin, cefoxitin, and cefotaxime and hence responsible for phenotypic extended-spectrum *β* -lactamases [31-32]. *β*-Lactamases encoded by *bla*_CTX-M_ and *bla*_TEM_ gene produced by *A. baumannii* strains are plasmid-mediated and could be responsible for overall and long-term survival. There are more than 40 CTX-M enzymes, which can be classified into five groups [33-34]. The *β*-lactamases from the TEM type are TEM-1- and TEM-2-derived enzymes. TEM-1 is the most crucial *β*-lactamase in Gram-negative bacteria. More than 130 TEM enzymes have been identified, which utilize a valuable method for distributing resistant genes [35-36]. In addition, the *bla*_AmpC_ and *bla*_TEM_ genes encode cephalosporinases. In a study, the latter was predominant in 100% of isolates, which have been shown to be inducible as concluded by [37]. *A. baumannii* produces intrinsic *β*-lactamases AmpC type cephalosporins expressed at low levels [38]. In this study, the *bla*_AmpC_ gene was present in the chromosomes of 24 isolates (24/27). The *bla*_AmpC_ gene was not detected in three isolates, P2, P12, and P48. Among them, P48 was susceptible to ceftazidime and showed 4 µg/mL MIC.

IS*AB-1* upstream of the *bla*_AmpC_ gene was found in 3 isolates that show MIC≥ 64 and produce no inhibition zone against ceftazidime. *A. baumannii* produces intrinsic *β*-lactamases such as an AmpC type which is expressed at low levels. Yet, when this sequence is located upstream of the *bla*_AmpC_ gene, enhanced expression occurred and provides resistance to third-generation cephalosporins [38-39]. A study showed that IS*AB-1* located upstream of *bla*_AmpC_ was observed in 48% of the *A. baumannii* isolates; the MIC was 64–256 *μ*g/mL [3]. However, IS*AB-1* is not present in the type strain ATCC 19606 [40].

Molecular epidemiology typing is a technique for detecting and tracking outbreaks of bacterial pathogens, as well as directing the spread of bacteria associated with hospital-acquired infections [13]. ERIC-PCR is a simple, rapid, and affordable method that has been conducted for clinical *A. baumannii* isolate distinction [31]. Using consensus primers, ERIC-PCR amplifies intervening fragments among highly conserved ERIC sequences. In this study, the dendrogram cluster analysis for the 27 strains distinguished six distinct clusters and three single patterns as a type strain. Among them, two significant clusters from a nosocomial point of view as source infection were identified to contain strains isolated from patients and the hospital environments. Cluster (*A*) was found to contain strains isolated from the patient in the male adult ward; P9, pediatric ward; P23, a strain isolated from CBU-oxygen mask; E1 and pediatric ward-oxygen ventilator machine; E58, whereas cluster (B) included strains isolated from the patient at male adult ward; P12, pediatric ward; P48 and a strain isolated from CBU-patient's bed; E4 and female adult ward-patient's bed; E19 (Table 6).

The multidrug resistance nature due to enzymatic mechanisms, especially plasmid-mediated resistance, which facilitate horizontal gene transfer among the isolates, may adversely affect and limit the medical treatments and hence the control of the nosocomial infections since the burn hospital suffers from endemic infections, especially those caused by *A. baumannii* [17].

Nosocomial infections represent a hazardous public health problem in all countries. It affects 5–15% of hospitalized inpatients in regular wards and more than 50% of ICU patients. Controlling and preventing nosocomial infections can be supported mainly by monitoring hospital settings, which allows for preventive and corrective actions through a better understanding of the microbial ecology [15]. While cross-contamination via hands is likely the highest risk, the risk of surface contamination should still be considered the main cause of infection as far as there are high chances of contamination of medical equipment which the patient may touch [41-42]. Biofilm-producing bacterial pathogens enable their survival on surfaces; hence, direct contact with these surfaces transmits most nosocomial infections [43]. Many nosocomial pathogens have been identified that are transmitted between inpatients and might survive in the environment for a long time and may persist on dry hospital inanimate surfaces under certain conditions [44-45]. Regularly used medical apparatus and joint surfaces could be a source of contamination by multidrug-resistant bacteria, especially intensive care units, which is more critical and poses an even more significant challenge [46].

Many studies have been established to identify the genetic relationship among nosocomial infections in different hospital settings worldwide: Saudi Arabia, Turkey, Iran, and China [13,14, 47,48]. They all concluded that the ERIC-PCR method is a simple, rapid typing technique with a level of discrimination and somehow equivalent to other methods such as PFGE and MLST and yielded almost the same results.

## 5. Conclusion

In conclusion, the emergence of XDR *A. baumannii* in the burn hospital is alarming, and the current study suggests high possibilities of *β*-lactamase genes dissemination between intra-inter wards and patients that might be closely associated with the high resistance levels. The dissemination of these strains among patients figures out the need for constant surveillance for applying adequate measures to avoid new nosocomial outbreaks caused by XDR *A. baumannii* strains. Wards environment directs inpatients to high risks for infections. ERIC-PCR provides a powerful, fast, accurate, and affordable technique for monitoring and controlling the nosocomial infection.

### 5.1. Limitations

Two classes (A and C) of ESBL encoding genes were evaluated, while other genes and their types are known which provide resistance to cephalosporins. Metallo-*β*-lactamase encoding genes (classes B and D) that provide resistance to carbapenems antibiotics were not evaluated. Finally, there is an inability to conduct gold standards and other advanced molecular methods to perform genotyping.

## Figures and Tables

**Table 1:** Primers and PCR settings used in this study.

Primer	Nucleotide sequence	Amplicon size (bp)	PCR condition
16s rRNA	F-TGGCTCAGATTGAACGCTGGCGGC	1500	Initial denaturation: 95 °C for 4 min, 35 cycles of denaturation: 95 °C for 40s, annealing: 64 °C for 40s, extension: 72 °C for 40s, and final extension: 72 °C for 5 min.
	R-TACCTTGTTACGACTTCACCCCA		
*bla*_Oxa-51_	F-TAATGCTTTGATCGGCCTTG	353	Denaturation: 94 °C for 5 min, 33 cycles of denaturation: 94 °C for 25s, annealing: 56.5 °C for 40s, extension: 72 °C for 50 s, and final extension: 72 °C for 6 min.
	R-TGGATTGCACTTCATCTTGG		
*bla*_SHV_	F-CTT TAT CGG CCC TCA CTC AA	237	Initial denaturation: 94 °C for 4 min, 30 cycles of denaturation: 94 °C for 60s, annealing: 57.3 °C for 40s, extension: 72 °C for 70s, and final extension: 72 °C for 10 min.
	R-AGG TGC TCA TCA TGG GAA AG		
*bla*_TEM-1_	F-CGC CGC ATA CAC TAT TCT CAG AAT GA	445	Initial denaturation: 94 °C for 4 min, 30 cycles of denaturation: 94 °C for 60s, annealing: 62.8 °C for 40s, extension: 72 °C for 70s, and final extension: 72 °C for 10 min
	R-ACG CTC ACC GGC TCC AGA TTT AT		
*bla*_CTX-M_	F-ATG TGC AGY ACC AGT AAR GTK ATG GC	593	Initial denaturation: 94 °C for 4 min, 30 cycles of denaturation: 94 °C for 60s, annealing: 68.6 °C for 40s, extension: 72 °C for 70s, and final extension: 72 °C for 10 min
	R-TGG GTR AAR TAR GTS ACC AGA AYC AGC GG		
*bla*_AmpC_	F-ACAGAGGAGCTAATCATGCG	1243	Initial denaturation: 94 °C for 4 min, 30 cycles of denaturation: 94 °C for 60s, annealing: 53.2 °C for 40s, extension: 72 °C for 60s, and final extension: 72 °C for 10 min
	R-GTTCTTTTAAACCATATACC		
ISAba1-F	F-CACGAATGCAGAAGTTG	1507	Initial denaturation: 95 °C for 5 min, 35 cycles of denaturation: 95 °C for 45 s, annealing: 52 °C for 50s, extension: 72 °C for 50 s, and final extension: 72 °C for 10 min
AmpC-R	R-GTTCTTTTAAACCATATACC		
-	TGAAGCTCCTGGGGATTCAC	-	Initial denaturation: 95 °C for 5 min, 30 cycles of: denaturation 95 °C for 60s, annealing: 40 °C for 60s, extension: 72 °C for 40s, and final extension: 72 °C for 5 min
	AAG TAA GTG ACT GGG GTG AGC G		

**Table 2:** Source of *A. baumannii* isolates from inpatients in different hospital wards.

Ward	Sample code
Male adult ward	P9
	P12
Female adult ward	P2
	P10
	P16
	P27
	P50
Pediatric ward	P8
	P23
	P48
	P49

**Table 3:** Source of *A. baumannii* isolates from environments in different hospital wards.

Ward	Sample code	Site of isolation
Critical burn unit-CBU	E1	Oxygen mask
	E2	Patient's cabinet surface
	E3	Patient's bed
	E4	IV stainless steel stand
Critical burn unit-office	E15	Medicine cabinet knob-1
	E16	Medicine cabinet knob-2
	E17	Office desk and chair
Female burn ward-section C	E18	Doorknob
	E19	Patient's bed
	E21	Doorknob
	E24	Toilet doorknob
	E26	Bathroom water mixer
Operation hall	E33	Bin
Pediatric ward 1	E53	Trolley-stainless steel-surface
	E54	IV stainless steel stand
	E58	Oxygen ventilator machine

**Table 4:** Susceptibility pattern of *A. baumannii* to different antimicrobial agents.

Antimicrobial classes	Antimicrobial agents	*A. baumannii* (*N* = 27)					
		S		I		R	
		n	%	n	%	n	%
Penicillins	Piperacillin	9	33.33			18	66.66
	Penicillin					27	100
*β*-Lactam-*β*-lactamase inhibitor	Ampicillin-sulbactam	9	33.33	6	22.22	12	44.44
Carbapenem	Meropenem	8	29.62			19	70.37
Cephalosporin	Cefotaxime	2	7.40	7	25.92	18	66.66
	Ceftazidime	9	33.33			18	66.66
	Cefazolin					27	100
	Cefoxitin					27	100
	Cefepime	7	25.92	2	7.4	18	66.66
Sulfonamides	Trimethoprim/Sulfamethoxazole	9	33.33	6	22.22	12	44.44
Aminoglycosides	Gentamicin	10	37.03			17	62.96
	Tobramycin	10	37.03	4	14.81	13	48.14
Fluoroquinolones	Levofloxacin	11	40.74	3	11.11	13	48.14
	Ciprofloxacin	11	40.74			16	59.25
Nitrofuran	Nitrofurantoin					27	100
Tetracyclines	Minocycline	27	100				
Chi-square		124.9					
*P* value		<0.0001					

**Table 5:** Resistant pattern XDR and MDR of *A. baumannii*.

Antimicrobial classes		Resistant pattern of *A. baumannii* (*N* = 27)				
		XDR (17/27)		MDR (10/27)		
		*N* = 16	*N* = 1	*N* = 8	*N* = 1	*N* = 1
Penicillins		R	R	R	R	R
*β*-Lactam-*β*-lactamase inhibitor		R	R	S	R	S
Carbapenem		R	R	S	R	S
Cephalosporins		R	R	R	R	R
Sulfonamides		R	R	S	R	S
Aminoglycosides		R	R	S	S	R
Fluoroquinolones		R	S	S	S	S
Nitrofurans		R	R	R	R	R
Tetracyclines		S	S	S	S	S
Sample source	Patient: N (%)	7 (44%)	1 (100%)	1 (12%)	1 (100%)	1 (100%)
	Environment: N (%)	9 (56%)		7 (88%)		

**Table 6:** Clusters of *A. baumannii* strains from patient and environment isolates by the ERIC-PCR method.

Cluster	Isolate code	Number of strains
A	P9, P23, E1, E58	4
B	P12, P48, E4, E19	4
C	E15, E17	2
D	E3, E16, E26, E53	4
E	E18, E21, E24, E33	4
F	P2, P8, P10, P16, P27, P50	6

## Data Availability

The Internet source data used to support the findings of this study are included within the References section. No other data were used for the study.
